# Rare Clinical Course of Immunoglobulin G4-Related Inflammatory Abdominal Aortic Aneurysm with Multiple Rare Complications

**DOI:** 10.1155/2019/8249061

**Published:** 2019-06-03

**Authors:** Yuji Naito, Tsukasa Miyatake, Manami Iwasaki, Atsushi Okuyama, Akio Takada, Koji Chiba, Masahiko Obata, Junichi Oba

**Affiliations:** ^1^Department of Thoracic Surgery, Asahikawa City Hospital, Asahikawa, Japan; ^2^Department of Cardiovascular Surgery, Hokko Memorial Hospital, Sapporo, Japan; ^3^Department of Radiation Medicine, Hokkaido University Graduate School of Medicine, Sapporo, Japan; ^4^Department of Internal Medicine, Fukagawa Municipal Hospital, Fukagawa, Japan; ^5^Department of Pathology, Asahikawa City Hospital, Asahikawa, Japan; ^6^Department of Hematology, Asahikawa City Hospital, Asahikawa, Japan; ^7^Department of Pathology, Asahikawa Red Cross Hospital, Asahikawa, Japan; ^8^Department of Cardiovascular Surgery, Hokkaido Medical Center for Child Health and Rehabilitation, Sapporo, Japan

## Abstract

Immunoglobulin G4- (IgG4-) related inflammatory abdominal aortic aneurysm (AAA) has been recognized as a manifestation of IgG4-related disease (IgG4-RD). We experienced one patient with multiple consecutive manifestations before and after endovascular stent grafting for IgG4-related inflammatory AAA (IAAA). A 71-year-old man was diagnosed with IgG4-RD due to increased IgG4 serum concentration, typical findings of parotid gland biopsy, and periaortitis in another hospital 2 years and 7 months before visiting our hospital. He came to our hospital because of abdominal pain and IAAA. He developed paraplegia after hospitalization and underwent endovascular stent grafting for the IAAA. About one month after stent grafting, he developed perforation of the sigmoid colon due to enteritis. He also had myocardial infarction. Finally, he died of intestinal bleeding. Here, we describe this case with rare, multiple, consecutive manifestations of IgG4-RD, some of which might be caused by IgG4-related IAAA or side effects of treatments rather than by IgG4-RD itself. We report this case because the clinical course seemed rare for IgG4-RD or IgG4-related IAAA. For treating IgG4-RD with IgG4-related IAAA, we should consider factors causing the symptoms and carefully select the proper treatment.

## 1. Introduction

Immunoglobulin G4-related disease (IgG4-RD) is a systemic condition with elevated serum IgG4 levels and infiltration of IgG4-positive plasma cells in organs such as the biliary tree, salivary glands, and retroperitoneum. IgG4-RD is commonly responsive to glucocorticoid therapy [1]. In the cardiovascular field, manifestations of IgG4-RD include retroperitoneal fibrosis and periaortitis. Recently, some inflammatory abdominal aortic aneurysms (IAAAs) have been considered to be related to IgG4-RD and are called IgG4-related IAAAs [2–4]. There are also some reports of coronary arteritis, though they are rare [5, 6]. We report the case of a patient who was diagnosed with IgG4-RD due to increased IgG4 serum concentration, parotitis, and periaortitis before visiting our hospital. He presented with abdominal pain caused by an IAAA. He had paraplegia, enteritis, and acute myocardial infarction before and after stent grafting. We report this case because the clinical course seemed rare for IgG4-RD or IgG4-related IAAA.

## 2. Case Presentation

**Figure 1:**
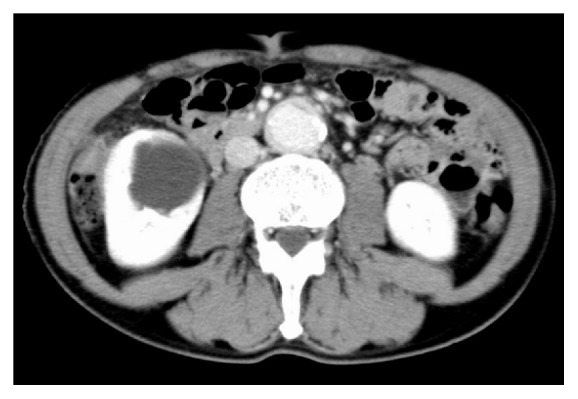
CT scan when the patient was first diagnosed with IgG4-RD with peritoneal fibrosis and periaortitis. CT, computed tomography; IgG4-RD, IgG4-related disease.

**Figure 2:**
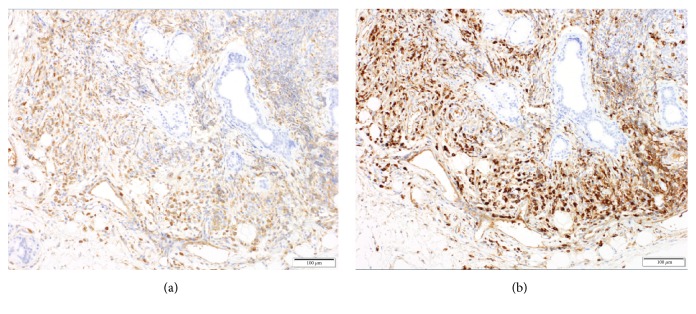
Pathology of the parotid gland at the diagnosis of IgG4-RD. (a) IgG staining and (b) IgG4 staining. There are many plasma cells stained with IgG (a) and 90% of them were positive for IgG4 (b). IgG4-RD, IgG4-related disease; IgG, immunoglobulin G.

A 71-year-old male patient visited another hospital due to abdominal pain 2 years and 7 months before visiting our hospital. He also had swelling of the lymph nodes in the neck and both inguinal regions. He had been diagnosed with peritoneal fibrosis, periaortitis, and bilateral parotitis based on a computed tomography (CT) scan (Figure 1) and physical examination findings. The serum level of IgG4 was 3260 mg/dl, and biopsy of the parotid gland showed infiltration of IgG4-positive plasma cells (Figure 2). He was diagnosed with IgG4-RD and was treated with prednisolone (PSL) 20 mg/day. He soon stopped taking PSL by his own judgment because he completely recovered from abdominal pain. He did not visit any hospital for more than 2 years.

**Figure 3:**
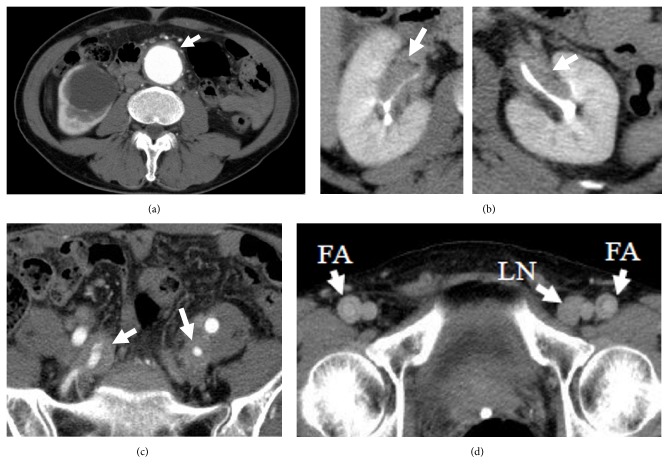
CT scan when the patient first came to our hospital. (a) The abdominal aorta was enlarged, and the adventitia was thickened. (b) Soft tissues around the ureters were thickened. (c) The tissue around the bilateral internal iliac arteries was thickened. (d) The adventitia of the bilateral femoral arteries was thickened and lymph nodes were swollen. CT, computed tomography; FA, femoral artery; LN, lymph node.

**Figure 4:**
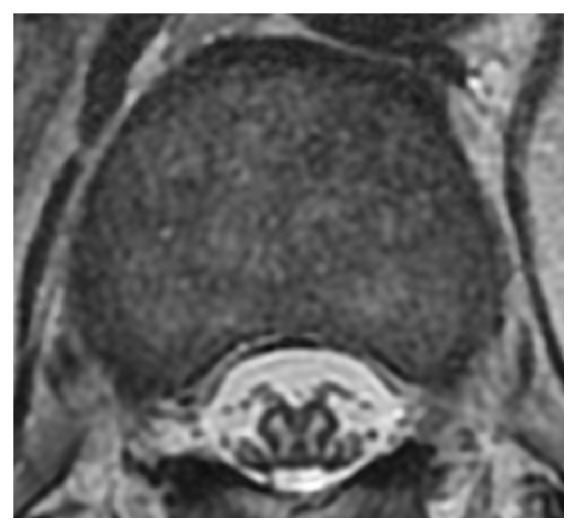
T2-weighted MRI at the T12 level showed bilateral hyperintensities in the anterior horns of the grey matter. MRI, magnetic resonance imaging.

He then visited our hospital due to abdominal pain since the last 2 months. A CT scan showed a 44 mm diameter AAA with thickened adventitia (Figure 3(a)). It was diagnosed as IAAA. The CT scan also showed thickening of tissue around the ureters (Figure 3(b)), internal iliac arteries (Figure 3(c)), and femoral arteries (Figure 3(d)). The lymph nodes were also swollen (Figure 3(d)). A blood test showed high serum levels of IgG (4225 mg/dl) and IgG4 (1890 mg/dl) (Table 1). IAAA was larger than before, but rupture or impending rupture was not detected in the CT scan image. Recurrence or aggravation of IgG4-RD was strongly suggested. The frequency of abdominal pain decreased after hospitalization, and we observed him with antihypertensive medicine treatment. On day 6, however, he had severe pain from the neck to the abdomen, and his systolic blood pressure rose to over 200 mmHg. Simultaneously, cyanosis and strong pain appeared in both lower limbs, and, thereafter, muscular strength of both lower limbs weakened. The manual muscle testing result of the iliopsoas muscle, quadriceps femoris, and anterior tibialis muscle was grade 0. He also had urinary retention. Based on magnetic resonance imaging (MRI) findings (Figure 4), the neurologist concluded that he had paraplegia from the 11th thoracic spinal cord level due to anterior spinal artery syndrome. The CT scan did not show aortic rupture. Soon after this episode, PSL 20 mg/day was restarted.

Even after restarting PSL treatment, he had abdominal pain several times and we could not completely exclude the possibility of the IAAA impending rupture. We performed endovascular stent grafting for the IAAA on day 13 after hospitalization. Under general anaesthesia, the bilateral inguinal portion was incised. The main body of the Excluder® (Japan GORE, Tokyo, Japan) was inserted and deployed through the left common femoral artery. Through the right common femoral artery, two stent graft limbs were inserted and deployed. Both internal iliac arteries were kept patent. Macroscopically, the adventitia of the right femoral artery was thickened. Microscopically, the intima and media showed atherosclerotic changes, without immunostaining of IgG4.

On day 6 after stent grafting, he had strong pain in both lower limbs and systemic cyanosis. His systolic blood pressure increased to over 200 mmHg. PSL dose was increased to 50 mg/day, and intravenous treatment of prostaglandin E1 was started.

On day 7 after stent grafting, he had bloody stools. Upper and lower intestinal endoscopy showed atrophic gastric mucosa, duodenal erosion, and ulceration and erosion from the sigmoid colon to the rectum. He was diagnosed with ischemic colitis. On day 34, he had strong pain in the abdomen and both lower limbs. A CT scan showed free air in the abdomen and emergent abdominal operation was performed. Macroscopically, he showed ischemic changes from the ileum to the upper rectum and perforation of the sigmoid colon. The intestine and peritoneum were strongly adhesive, and we could not observe some IgG4-RD related organs, such as pancreas and biliary tract. The lymph nodes were not swollen in the mesentery. The wide portion from the ileum to the rectum was resected and colostomy was performed. The pathology of the intestine showed cytomegalovirus colitis and ganciclovir was soon started. He was transferred for rehabilitation to another hospital on day 124 after admission and on day 111 after stent grafting.

**Figure 5:**
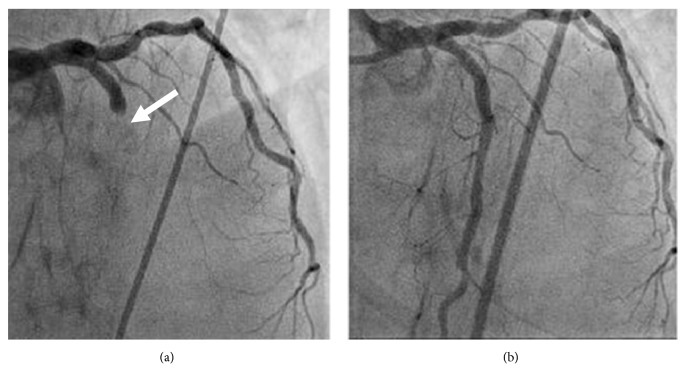
Coronary angiogram at the time of myocardial infarction (a) and after endovascular intervention (b). Angiogram at the time of myocardial infarction (a) showing occlusion of the left anterior descending artery with thrombus.

**Figure 6:**
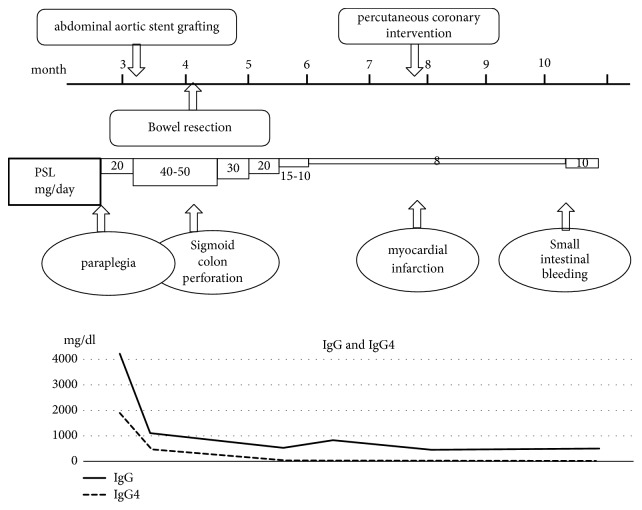
Progress of manifestations, IgG, IgG4, and treatments. IgG and IgG4 serum levels became normal 1 month after PSL administration. PSL, prednisolone; IgG, immunoglobulin G.

About one month later, he was transferred to our hospital again with acute myocardial infarction. Aspiration of the thrombus and stenting for the occluded left descending artery were performed (Figure 5). Three months later, he came to our hospital again with intestinal haemorrhagic shock. He died of massive bleeding from colostomy. Autopsy revealed wide range of intestinal erosion with ischemia (Figure 6). IgG4 staining and cytomegalovirus test results of the intestine were negative.

## 3. Discussion

We reported the case of a patient with rare, multiple, consecutive complications of IgG4-related IAAA. IgG4-RD is a disease concept proposed by Hamano et al., based on the relationship between autoimmune pancreatitis and IgG4 [1]. Its manifestations in organs such as the lacrimal gland, salivary gland, respiratory system, digestive system, hepatobiliary system, renal urinary system, endocrine system, nervous system, lymphoid system, musculoskeletal system, and cardiovascular system were recognized later on [7]. According to the IgG4-RD diagnostic criteria 2011 [8], our patient was diagnosed with IgG4-RD in another hospital. High IgG4 serum level, parotitis with IgG4-positive plasma cells, periaortitis, and effectiveness of PSL were typical for IgG4-RD. When he first came to our hospital, he also had typical manifestations of IgG4-RD. The high level of serum IgG4, IAAA with abdominal pain, periarteritis of the iliac arteries and femoral arteries, and thickness of the tissue around the ureter indicated recurrence or aggravation of IgG4-RD. He first had 20 mg/day of PSL in another hospital and ceased it by himself. In Japanese prospective studies [9, 10], the efficacy of the initial and maintenance therapy of PSL to IgG4-RD was shown. In this case, the cessation of PSL could be a main cause of recurrence or aggravation. In the same studies, the authors administered 30 mg/day or 0.6 mg/kg/day of the initial dose of PSL, though our patient was administered 20 mg/day of it as the initial and restart doses. In another study [11], 0.4-0.69 mg/kg/day of the initial dose was reported to be adequate to prevent recurrence. The dose of 20 mg/day was selected because the patient's weight was 50 kg and his physicians wanted to give the minimum effective dose in another hospital. We chose the same dose at the restart. However, 30 mg/day might have been a better dose as it showed clear evidence in prospective studies.

The manifestations or diseases that developed after he came to our hospital (paraplegia from anterior spinal artery syndrome, myocardial infarction, and enteritis) seemed to be atypical of IgG4-RD. We cannot be sure whether they were related to IgG4-RD.

Paraplegia is very rare with IgG4-RD, but there are some reports of paraplegia from sclerosing pachymeningitis [12, 13]. We carefully examined the MRI findings of this patient, but there were no findings of spinal cord compression or sclerosing pachymeningitis. The neurologist diagnosed anterior spinal artery syndrome from the bilateral hyperintensities in the anterior horns of the grey matter on T2-weighted MRI. To the best of our knowledge, there are no reports on anterior spinal artery syndrome with IgG4-RD. IgG4-related IAAA might be related to paraplegia because there was a report on paraplegia with AAA [14].

Myocardial infarction is also rare in IgG4-RD, but reports exist [6, 15]. Sakamoto et al. reported the progression of coronary disease in proportion to the serum level of IgG4 [16]. These reports indicated that myocardial infarction might be one of the manifestations of IgG4-RD. When we examined retrospectively the CT image taken at the first admission of this patient, we found right coronary periarteritis, although the occluded artery was the left coronary artery. At the time of the acute myocardial infarction, the serum level of IgG4 was normal. Hence, it is unclear whether his myocardial infarction was related to IgG4-RD.

Regarding his enteritis, he had ischemic and cytomegalovirus enteritis. First, he was diagnosed with ischemic colitis with endoscopy, and, at the time of perforation of the sigmoid colon, the pathology showed cytomegalovirus enteritis. There was a report on cytomegalovirus enteritis along with ischemic enteritis [17], as well as a report on cytomegalovirus enteritis with a manifestation similar to that of ischemic enteritis [18]. It is unclear whether our patient had ischemic colitis at the early phase, but it is clear that he was complicated with it just before he died. With regard to ischemic colitis, we could not exclude atheromatous embolization by stent grafting or IgG4-related IAAA itself, which has features of AAA. There is a report stating that 1.4% of cases of endovascular treatment for AAA complicated acute bowel ischemia [19]. Regarding cytomegalovirus enteritis, PSL definitely influenced its occurrence.

One of the most important factors that influenced atypical manifestations of IgG4-RD was PSL. Glucocorticoids are associated with an increased risk of myocardial infarction [20–22]. As previously mentioned, the patient's cytomegalovirus enteritis was related to PSL.

Another factor might be arterial spasms. He had high blood pressure, systemic pain rather than local pain, and systemic cyanosis when his condition worsened. We believe that his coronary artery, intestinal artery, limb artery, and spinal cord artery were spastic and caused the ischemia of the related organs. We administered nitric acid medicines, calcium antagonists, and prostaglandin E1, but the effects were not clear. We believe that the vascular spasms were related to his clinical conditions, although we could not find studies on the relationship between IgG4-RD and vascular spasms.

Another strong possibility is that he had atheromatous embolization, which could explain his enteritis, myocardial infarction, pain in the bilateral lower limbs, and anterior spinal artery syndrome. IgG4-related IAAA, which does not have only features of IgG4-RD but also those of AAA, might have caused or been related to atheromatous embolization and some complications.

We thought of other differential diagnoses, but his conditions did not fit into any of them. When he came to our hospital, antinuclear antibody was negative, white blood cell count was 8840 *μ*L, C-reactive protein level was 1.18 mg/dl (Table 1), and he had no fever. Infection, other vasculitis, and autoimmune diseases were ruled out or not diagnosed. Bone marrow puncture did not show the finding of myeloma. CT scan, MRI, gastrointestinal endoscopy, and autopsy did not show the findings of malignant tumors.

Thus, we reported the case of a patient with IgG4-related IAAA who had parotitis, paraplegia, enteritis, and myocardial infarction before and after stent grafting. The clinical course seemed rare for IgG4-RD. We speculate that some symptoms or diseases were caused by atheromatous embolization which might be related to IAAA, corticosteroids, or arterial spasms rather than IgG4-RD itself. During the treatment of IgG4-RD, especially if complicated with IgG4-related IAAA, we should consider if the symptoms are caused by IgG4-RD or other factors and carefully select the proper treatment.

## Figures and Tables

**Table 1:** Laboratory test results on admission.

Variable (unit)	value
White blood cells (*μ*L)	8840
Segmented cells (%)	61
Eosinophils (%)	20.9
Basophils (%)	1.1
Monocytes (%)	2.9
Lymphocytes (%)	14.1
Hemoglobin (g/dl)	11.7
Platelets (K/*μ*L)	359
Total protein (mg/dl)	9.0
Albumin (mg/dl)	2.7
Albumin/Globulin	0.43
IgG (mg/dl)	4225
IgG4 (mg/dl)	1890
C-reactive protein (mg/dl)	1.18
Sedimentation rates (mm/1 hour)	122
Total cholesterol (mg/dl)	161
Triglycerides (mg/dl)	116
Hemoglobin A1c (%)	5.5
TPLA	(-)
Hepatitis B surface antigen	(-)
Hepatitis C antibody	(-)
Anti-nuclear antibody	(-)
Anti-SS-A antibody	(-)
Anti-SS-B antibody	(-)
APTT (seconds)	27.3
PT-INR	1.04
Fibrinogen (mg/dl)	377.5

TPLA, *Treponema pallidum* latex agglutination; APTT, activated partial thromboplastin time; PT-INR, prothrombin time-international normalized ratio.
